# Histone Deacetylase (HDAC) 1 Controls the Expression of Beta Defensin 1 in Human Lung Epithelial Cells

**DOI:** 10.1371/journal.pone.0050000

**Published:** 2012-11-20

**Authors:** Kimberley Kallsen, Ellen Andresen, Holger Heine

**Affiliations:** Division of Innate Immunity, Research Center Borstel, Airway Research Center North, Member of the German Center for Lung Research, Borstel, Germany; University Hospital Schleswig-Holstein, Germany

## Abstract

Deregulation of the expression human beta defensin 1 (DEFB1), an antimicrobial peptide, has been implicated in the pathogenesis of COPD and asthma. Since the molecular mechanisms that regulate DEFB1 gene expression are widely unknown, the epigenetic processes involved in the regulation of the constitutive expression of DEFB1 in lung epithelial cells (A549) were investigated. The data demonstrate that histone deacetylases (HDACs) participate in the regulation of DEFB1 gene expression. Inhibition of the class I HDACs, HDACs 1-3, increases DEFB1 gene expression in A549 cells. Chromatin immunoprecipitation (ChIP) assays revealed that the inhibition of the class I HDACs also results in modifications of the chromatin at the *DEFB1* promoter. Histone modifications, histone H3 acetylation and H3K4 trimethylation, that are associated with transcriptional activation, were found to increase after inhibition of HDACs 1-3. Finally, RNAi knockdown experiments identified HDAC1 as the sole HDAC responsible for maintaining the constitutive level of *DEFB1* transcription. Taken together, our data reveal epigenetic mechanisms which are the basis of the maintenance of the constitutive gene expression of human beta defensin 1.

## Introduction

Human beta defensins are 3–5 kDa polycationic peptides that are known for their antimicrobial activity against bacteria, fungi and viruses [1]. In addition, defensins are chemotactic attractants for immature dendritic cells and memory T cells [2]. Defensins also stimulate mitosis in fibroblasts and epithelial cells [3]. Thus, they play important roles in innate and adaptive immunity.

Beta defensin 1 (DEFB1) was the first human beta defensin isolated [4] and it is expressed not only throughout the respiratory epithelia [5] but also other epithelia and immune cells [4], [6], [7]. Polymorphisms of the *DEFB1* gene are associated with several diseases including chronic obstructive pulmonary disease (COPD) [8], oral Candida infections [9], asthma [10], atopic dermatitis [11], and periodontitis [12]. In addition, stage-dependent upregulation of *DEFB1* expression has been observed in the lungs of patients diagnosed with COPD [13]).

*DEFB1* is usually constitutively expressed [14], but some exceptions have been described, as in LPS- or IFN-γ-stimulated monocytes [15], uterine epithelial cells treated with TLR3 agonists [16], pulmonary epithelial cells stimulated with cell wall components from mycobacteria [17] and a kidney cell line stimulated with LPS and proinflammatory cytokines [18]. However, DEFB1 is thought to be a basal host defense molecule in the absence of injury or inflammation [19]. Otherwise, little is known about the regulation of DEFB1 gene expression. Because of its constitutive expression, epigenetic mechanisms might play an important role. In addition to the involvement of *DEFB1* polymorphisms in the pathogenesis of COPD, a deregulation of histone deacetylases (HDACs) has been observed in patients diagnosed with COPD [20].

HDACs function in histone deacetylation and work in opposition to the histone acetyltransferases (HATs) that increase histone acetylation. Together, these proteins function to regulate chromatin accessibility as HATs generally facilitate transcriptional activation whereas the antagonistic HDACs repress transcription [21]. Based on these observations we wanted to study the role of histone acetylation in the regulation of DEFB1 gene expression.

The results of the present study showed that HDAC1 controls the expression of DEFB1 in lung epithelial cells by regulating transcriptional accessibility at the *DEFB1* promoter.

## Results

### Inhibition of HDACs with the Pan-HDAC Inhibitor Trichostatin A Increases DEFB1 Gene Expression and Histone H3 Acetylation at the DEFB1 Promoter

**Figure 1:**
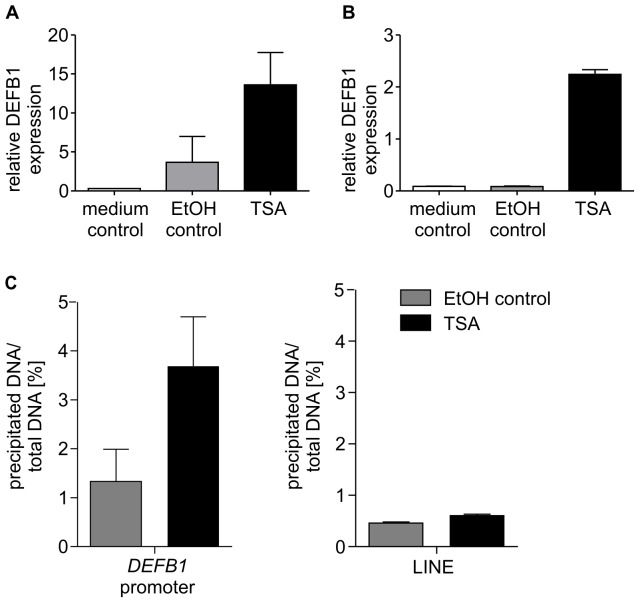
Modulation of DEFB1 gene expression and histone H3 acetylation by the HDAC inhibitor TSA. The human lung epithelial cell lines A549 (A) and NCI-H727 (B–C) were treated with the HDAC inhibitor trichostatin A for 24 h. (A–B) mRNA expression of *DEFB1* was analyzed using quantitative Real-Time-PCR. The measured levels of mRNA were normalized to *PBG-D* mRNA levels. (C) ChIP analysis was performed using anti-acetyl-Histone H3 antibody. Isolated DNA was quantified using quantitative RT-PCR. Bars represent means with SD of three independent experiments. LINE: long interspersed nuclear element.

The constitutive gene expression of DEFB1 suggests a regulation by epigenetic mechanisms. Both, *DEFB1* polymorphisms [8] and HDAC deregulation [20], are associated with the pathogenesis of COPD. Thus, we hypothesized that DEFB1 gene expression is regulated by HDACs and analyzed the effect of the broad-spectrum HDAC inhibitor trichostatin A on DEFB1 gene expression. Treatment with trichostatin A for 24 h increased DEFB1 gene expression in two different lung epithelial cell lines, A549 (Fig. 1A) and NCI-H727 (Fig. 1B). Due to the unspecific effects that can be induced by trichostatin A and which may influence several cellular processes, we additionally analyzed histone H3 acetylation at the *DEFB1* promoter. Treatment with trichostatin A induced a 2.8 fold increase in histone H3 acetylation at the *DEFB1* promoter (−224 to −373) (Fig. 1C) whereas histone H3 acetylation at the transcriptionally inactive retrotransposon LINE was not altered.

### Inhibition of HDACs 1-3 Enhances DEFB1 Gene Expression in Human Lung Epithelial Cells

**Figure 2:**
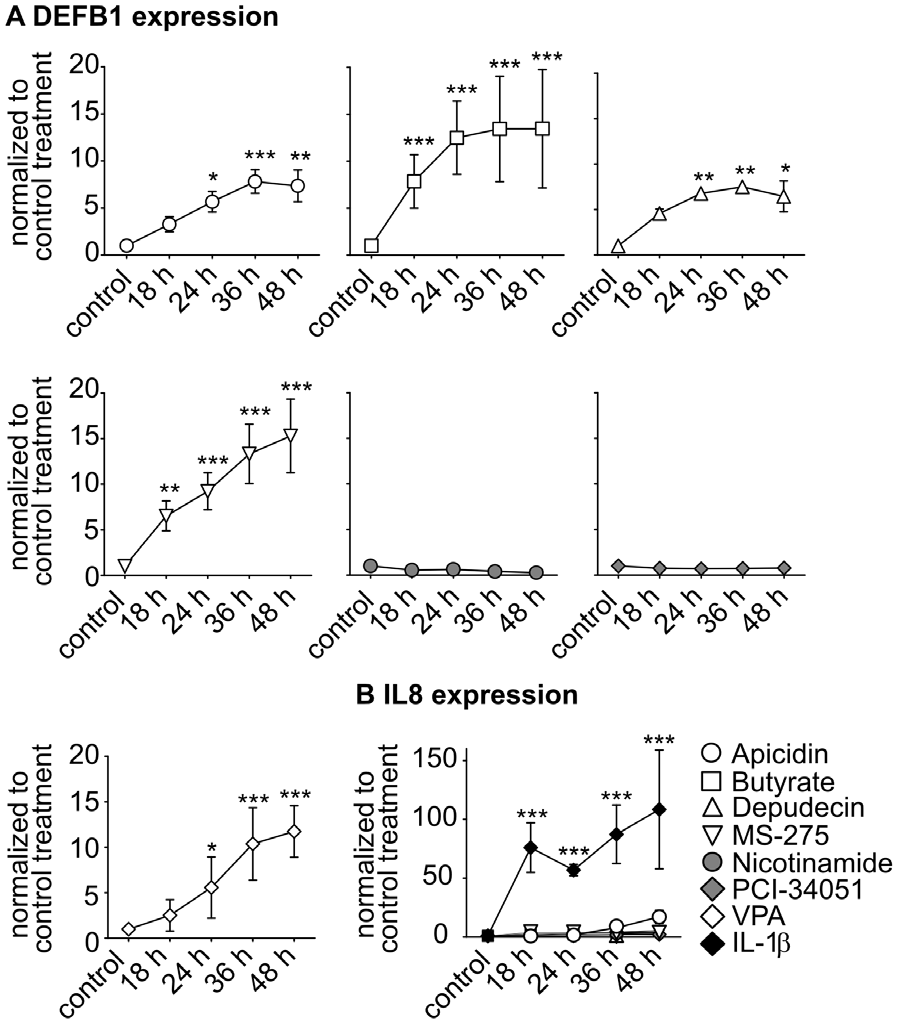
Modulation of DEFB1 gene expression by HDAC inhibitors. The human lung epithelial cell line A549 was treated with HDAC inhibitors and IL-1β for 18 to 48 h. mRNA expression of *DEFB1* (A) and *IL8* (B) was analyzed using quantitative Real-Time-PCR. The measured levels of mRNA were normalized to *TBP* mRNA levels. *DEFB1/IL-8* mRNA expression was normalized to control treatment. Bars represent means with SD of three independent experiments (*: p<0.05; **: p<0.01; ***: p<0.001).

To investigate the role of HDACs in the transcriptional regulation of constitutive DEFB1 gene expression in lung epithelial cells in more detail and to identify the responsible HDAC, A549 cells (a human lung epithelial cell line) were treated with different HDAC inhibitors with increasing specificity. The inhibitors used were specific for the HDAC subgroups (s. also table 1): nicotinamide inhibits Class III HDACs (SIRT1-7) [22] while butyrate and valproic acid both inhibit Class I (HDACs 1-3 and HDAC8) and Class IIa HDACs (HDACs 4, 5, 7 and 9) [23], [24]. In addition, apicidin and depudecin inhibit Class I HDACs, [25], [26] MS-275 inhibits HDACs 1-3 [27] and PCI-34051 inhibits HDAC8 [28]. Apicidin, butyrate, depudecin, MS-275 and valproic acid increase DEFB1 gene expression that increases with time. Neither nicotinamide nor PCI-34051 modulated DEFB1 gene expression (Fig. 2A). To determine if the HDAC inhibitors induced general activation of the cells, IL8 gene expression was measured because IL8 is commonly synthesized by epithelial cells following activation. Stimulation of the cells with the proinflammatory cytokine IL-1β was used as a positive control. Whereas none of the HDAC inhibitors increased IL8 gene expression in A549 cells IL-1β showed typical induction of IL-8 mRNA expression (Fig. 2B).

**Table 1:** Specificity of HDAC inhibitors.

HDAC inhibitor	target HDACs
trichostatin A	Class I (HDAC1-3 and HDAC8)
	Class IIa HDACs (HDAC4, 5, 7, and 9)
	Class IIb (HDAC6 and 10)
	Class IV (HDAC11)
Nicotinamide	Class III HDACs (SIRT1-7)
butyrate/valproic acid	Class I (HDAC1-3 and HDAC8)
	Class IIa HDACs (HDAC4, 5, 7, and 9)
apicidin/depudecin	Class I (HDAC1-3 and HDAC8)
MS-275	HDAC1-3
PCI-34051	HDAC8

These results suggested that HDACs were involved in the maintenance of the constitutive expression of DEFB1 in lung epithelial cells. Considering the different specificities of the HDAC inhibitors, the HDAC responsible for the transcriptional repression of DEFB1 is probably HDAC1, HDAC2, or HDAC3.

### Inhibition of HDACs Induces a Transcriptionally More Active Chromatin Status at the DEFB1 Promoter

**Figure 3:**
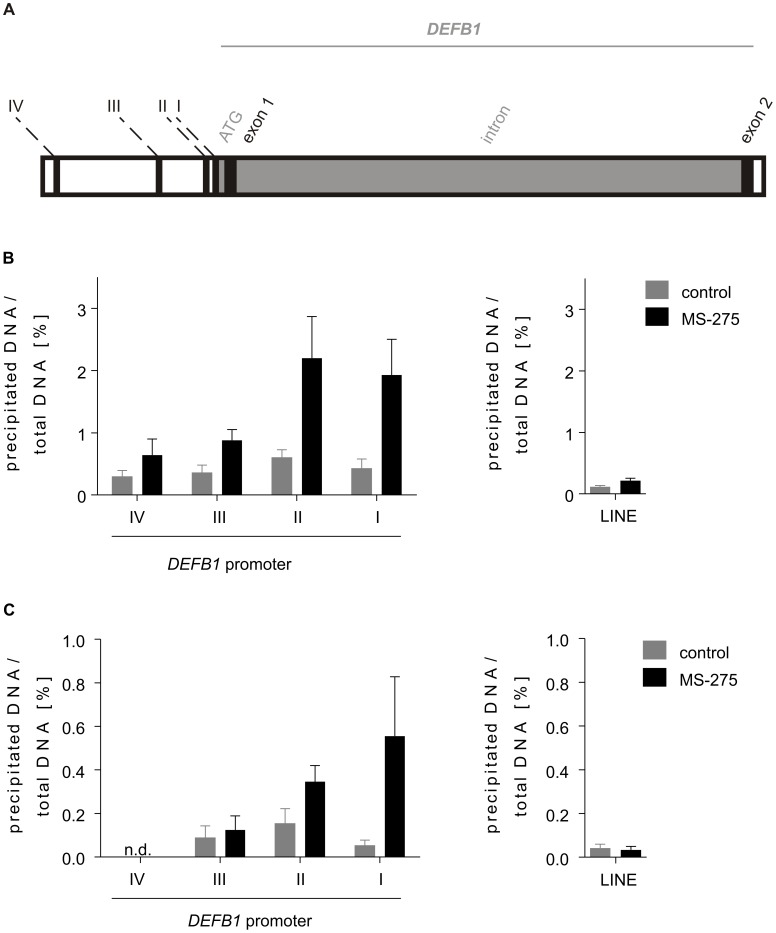
The HDAC inhibitor MS-275 increases H3 acetylation and H3K4 trimethylation at the *DEFB1* promoter. (A) DEFB1 gene with promoter region including analysis sites I-IV. I: −186 – −250, II: −347 – −406, III: −1002 – −1061, IV: −2436 – −2499. (B–C) A549 cells were treated with HDAC inhibitor MS-275 for 36 h. ChIP analysis was performed using anti-acetyl-Histone H3 (B) and anti-trimethyl-H3K4 (C) antibodies. Isolated DNA was quantified using quantitative RT-PCR. Bars represent means with SEM of three independent experiments. LINE: long interspersed nuclear element.

ChIP analysis was used to investigate whether inhibition of HDACs influences the structure of chromatin at the *DEFB1* promoter or if HDACs affect DEFB1 gene expression indirectly by modulating regulatory proteins that are substrates of HDACs. Therefore, we analyzed if treatment of A549 cells with the HDACs 1-3 inhibitor MS-275 modulates histone modifications at the *DEFB1* promoter. Four positions within the *DEFB1* promoter region were analyzed. These were named positions I to IV, with position I being the closest to the start of mRNA synthesis (Fig. 3A). Two histone modifications, the acetylation of histone H3 and the trimethylation of lysine 4 at histone 3 (H3K4), that are associated with transcriptionally active chromatin [29] were increased at the *DEFB1* promoter after treatment with MS-275 for 36 h (Fig. 3B–C). This increase in transcriptionally active chromatin correlated with the proximity to the transcription start point. Additionally, H3 acetylation and H3K4 trimethylation of the LINE retrotransposon, which is usually transcriptionally inactive, were used to assess the specificity of the analysis. Neither H3 acetylation nor H3K4 trimethylation of LINE were enhanced after treatment with the HDAC inhibitor MS-275.

The results suggested that the inhibition of HDACs 1-3 enhanced DEFB1 gene expression by increasing transcriptional activity at the *DEFB1* promoter.

### Knockdown of HDAC1, but not HDAC2 or HDAC3, Increases DEFB1 Gene Expression in A549 Cells

**Figure 4:**
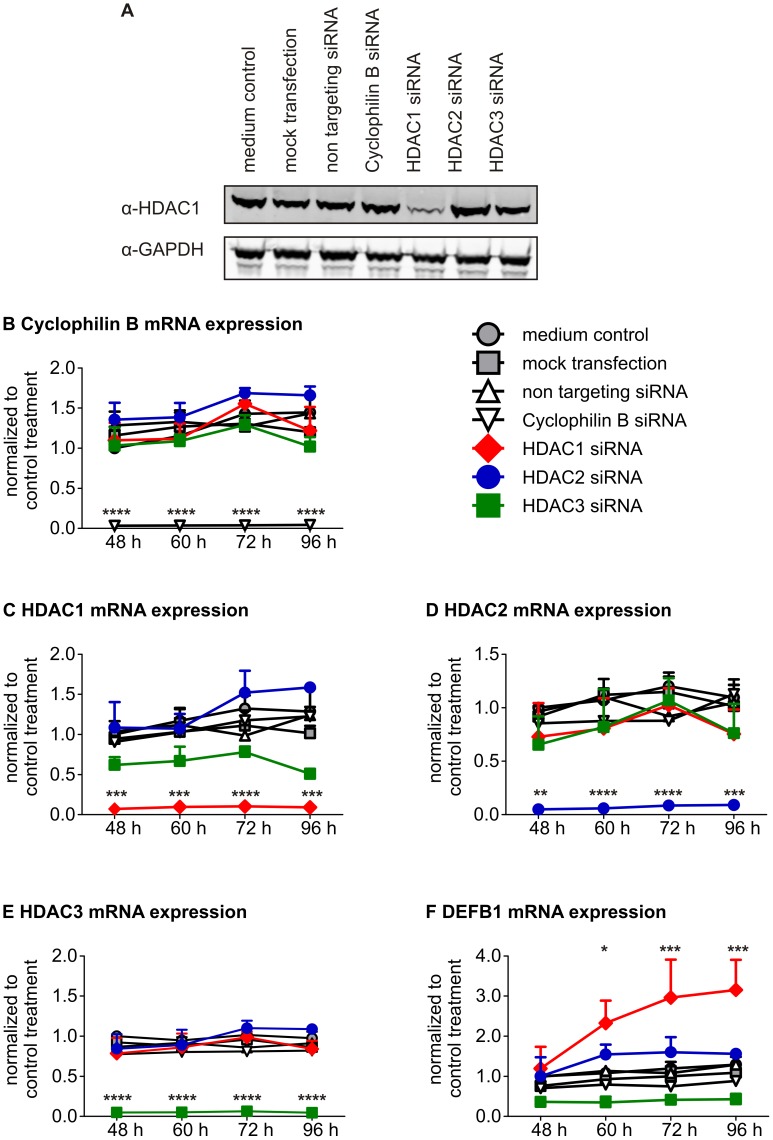
Knockdown of HDAC1 increases DEFB1 gene expression. A549 cells were transfected with HDAC and control siRNAs for 48 to 96 h. (A) Cells were lysed 48 h after transfection and Western Blot analysis was performed with an anti-HDAC1 antibody. Data are representative of three independent experiments. (B–F) mRNA expression of *Cyclophilin B* (B), *HDAC1* (C), *HDAC2* (D), *HDAC3* (E) and *DEFB1* (F) was analyzed using quantitative Real-Time-PCR. The measured levels of mRNA were normalized to *TBP* mRNA levels. Bars represent means with SEM of three independent experiments (*: p<0.05; **: p<0.01; ***: p<0.001, ****: p<0.0001).

To determine whether HDAC1, HDAC2 or HDAC3 were responsible for the transcriptional repression of *DEFB1* in lung epithelial cells, siRNA was used to knock down expression of the HDACs in A549 cells. Mock transfection, non-targeting siRNA, and siRNA targeting the housekeeping gene cyclophilin B were used as controls (Fig. 4B-F). Each specific siRNA inhibited expression of its target mRNA (Fig. 4B–E) and the HDAC1 siRNA repressed HDAC1 protein expression (Fig. 4A). The knockdown of cyclophilin B, HDAC2, or HDAC3 did neither increase DEFB1 gene expression nor did the HDAC3 siRNA significantly reduce DEFB1 gene expression. Only inhibition of HDAC1 significantly increased DEFB1 gene expression, starting at 60 h after transfection (Fig. 4F). These data showed that only HDAC1, but not HDAC2 or HDAC3, was involved in the transcriptional repression of *DEFB1* in A549 cells.

## Discussion

The aim of our experiments was to test whether the constitutive gene expression of the antimicrobial peptide DEFB1 was controlled by epigenetic factors and to study the underlying molecular mechanisms. Taken together, our data suggest that HDAC1 plays an important role in the maintenance of the constitutive expression of *DEFB1* in lung epithelial cells.

HDACs are important enzymes in the regulation of transcription. Since they are involved in multiple cellular processes like proliferation and survival, use of HDAC inhibitors such as TSA became an important strategy in the development of cancer therapy during the last years [30]. Furthermore, HDACs play a role in inflammatory disorders [31], [32]. Modulation of the dynamics between HDACs and the antagonizing HATs may lead to new anti-inflammatory treatments [33]. Presumably the best studied HDAC inhibitor is trichostatin A (TSA), a potent inhibitor of all class I and class II HDACs [34], [35]. However, using a broad-spectrum inhibitor of HDACs also bears the potential of unwanted side-effects, potentially contraindicating therapeutic use of such inhibitors. Thus, the interest is shifting towards the characterization of more specific inhibitors to selectively address individual HDACs and target distinct pathways [36].

In order to identify the HDAC that might be responsible for the repression of an elevated DEFB1 gene expression, we used HDAC inhibitors with increasing specificity and, after narrowing down the effect to HDACs 1-3, additionally siRNA to address these HDACs individually. HDAC inhibition led to a much stronger increase in DEFB1 gene expression than the RNAi experiments, suggesting a long half-life of the HDAC1 protein.

The upregulated *DEFB1* mRNA expression after treatment with the HDAC inhibitors could either be a direct (at the level of histones) or an indirect effect, since the function of a number of non-histone transcription factors and co-regulators, such as p53, hsp90, importin-α, and NF-κB, can also be modulated by acetylation [37]. However, inhibition of HDACs 1-3 increased two histone modifications at the *DEFB1* promoter that are associated with transcriptionally active chromatin [29]; the acetylation of histone H3 and the trimethylation of lysine 4 at histone 3 (H3K4). The increased acetylation of H3 is a strong indication for a direct effect of the HDAC inhibitor. The enhanced H3K4 trimethylation was probably a downstream consequence of transcriptional activation initiated by this change in the acetylation status. Thus, the data strongly suggest that knockdown/inhibition of HDAC1 enhanced DEFB1 gene expression by increasing transcriptional activity at the *DEFB1* promoter.

We hypothesize that under physiological conditions basal constitutive DEFB1 gene expression is a consequence of a particular epigenetic profile. This epigenetic profile is regulated by a protein complex which includes HDAC1 and the mechanism of regulation is based on the dynamic interplay of HDACs and HATs. Under certain pathogenic conditions such as COPD, the activity of HDACs has been shown to be reduced [20], conceivably because of cigarette smoke, which is the most frequently mentioned risk factor for COPD [38] and is known to affect the activity and stability of HDACs [39]. Our data suggest that in consequence of the reduced HDAC activity, acetylation at the *DEFB1* promoter increases. This is probably followed by relaxation of the chromatin structure that exposes additional transcription factor binding sites and thereby enables enhanced DEFB1 gene expression. Recently, Peyrin-Biroulet et al. [40] showed that peroxisome proliferator-activated receptor gamma (PPARγ) is involved in the maintenance of DEFB1 expression in the colon by binding directly to the *DEFB1* promoter. Since PPARγ is also expressed in the lung epithelium and lung epithelial cell lines, one might speculate that HDAC1 is involved in the regulation of PPARγ expression and/or signaling and therefore, both proteins cooperatively regulate DEFB1 expression.

High amounts of DEFB1 might contribute to the pathogenesis of COPD. The chemotactic activity of DEFB1 on immature dendritic cells and T memory cells [2] could play a role in the chronic inflammation detected in the airways of patients diagnosed with COPD [41], [42]. In summary, these results provide new insights into the epigenetic regulation of DEFB1 gene expression that may help to define new targets for therapeutic approaches for DEFB1-associated diseases [43].

## Materials and Methods

### Cells and Media

Both A549 (lung epithelial cells) and NCI-H727 cells (bronchial epithelial cells) were obtained from ATCC (Manassas, VA, USA). Cell lines were cultured in D-MEM (A549) or RPMI 1640 (NCI-H727, both Invitrogen) with 10% FCS, 100 U/ml penicillin (PAA) and 100 µg/ml streptomycin (PAA). A549 cultures were passaged twice a week with a density of 0.9 × 10^6^ cells per 75 cm^2^ area flask. NCI-H727 cultures were passaged once a week with a density of 1×10^6^ cells per 75 cm^2^ area flask.

### HDAC Inhibition

The HDAC inhibitors apicidin, depudecin, MS-275, nicotinamide and valproic acid were purchased from Enzo Life Sciences, butyrate and PCI-34051 were from Sigma and trichostatin A from Cell Signaling. A549 (2×10^5^) and NCI-H727 (5×10^5^) cells were seeded into 6-well plates the day before inhibitor treatment. HDAC inhibitors were used in the following concentrations and later compared to the stated control treatments: apicidin (1 µM/0.2% DMSO, Sigma), depudecin (10 µM/0.2% DMSO), MS-275 (1,5 µM/0.2% DMSO), nicotinamide (30 mM/3.4% aqua ad iniectabilia, Braun), valproic acid (7.5 mM/3.4% aqua ad iniectabilia), butyrate (5 mM/0.2% DMSO), PCI-34051 (10 µM/0.2% DMSO), trichostatin A (400 nM/0.01% ethanol, Merck). In preliminary experiments different concentrations of the HDAC inhibitors were used to identify the appropriate concentrations. For control treatment, 1 ng/ml recombinant human IL-1β (GIBCO) was used. At 18, 24, 36, and 48 h after inhibitor treatment, cells were lysed, total RNA was isolated (Absolutely RNA® Miniprep Kit, Agilent Technologies) and cDNA was synthesized (SuperScript III Reverse Transcriptase, Invitrogen).

### siRNA

The day before transfection, A549 cells were seeded into 6-well plates. The cell number was adjusted so that there were approximately 1×10^6^ cells at the end of the experiment. DharmaFECT1 transfection reagent and 100 nM siRNAs (both from Thermo Fisher Scientific) were used to transfect the cells. The following siRNAs were used: ON-TARGETplus Non-targeting Pool (D-001810-10), Human PPIB ON-TARGETplus SMARTpool (L-004606-00), Human HDAC1 ON-TARGETplus SMARTpool (L-003493-00), Human HDAC2 ON-TARGETplus SMARTpool (L-003495-00), Human HDAC3 ON-TARGETplus SMARTpool (L-003496-00). For RT-PCR analysis, cells were lysed 48, 60, 72, and 96 h after transfection, total RNA was isolated (Absolutely RNA® Miniprep Kit, Agilent Technologies), and cDNA was synthesized (SuperScript III Reverse Transcriptase, Invitrogen). For western blotting, cells were lysed in Laemmli Buffer 48 h after transfection.

### Quantitative RT-PCR

Quantitative RT-PCR was performed using the LightCycler 480 SYBR Green I Master (Roche) and the LightCycler 480 Probes Master (HDAC3 mRNA analysis) (Roche). For amplification and detection, LightCycler 480 (Roche) was used and the analysis was performed using LightCycler 480 Software (Roche). Using relative quantification, the measured levels of mRNA were normalized to either Porphobilinogen deaminase (PBGD) or TATA box binding protein (TBP) mRNA levels. This housekeeping gene was chosen using the geNorm software. The following primers were used: Cyclophilin B: GGAGATGGCACAGGAGGAA, TCTCCACCTTCCGCACC; DEFB1: TGTCTGAGATGGCCTCAGGT, GGGCAGGCAGAATAGAGACA; HDAC1: CCAAGTACCACAGCGATGAC, TGGACAGTCCTCACCAACG; HDAC2: TGAAGGAGAAGGAGGTCGAA, GGATTTATCTTCTTCCTTAACGTCTG; HDAC3: CACCATGCCAAGAAGTTTGA, CCCGAGGGTGGTACTTGAG; IL8: TTGCCAAGGAGTGCTAAAGAA, CAACCCTACAACAGACCCACAC; PBG-D: AACCCTGCCAGAGAAGAGTG, AGCCGGGTGTTGAGGTTT; TBP: GCTGGCCCATAGTGATCTTT, TCCTTGGGTTATCTTCACACG. To account the primer efficiency, external standard curves were used.

### Western Blotting

Cell lysates were resolved by electrophoresis in NuPAGE 4–12% Bis-Tris gels (Invitrogen) and transferred to PVDF membranes. After blocking in blocking solution (5% low fat dry milk (Saliter) in PBS, 0.1% Tween 20 (Honeywell Specialty Chemicals)) membranes were incubated with 0.4 µg/ml anti-GAPDH (clone 6C5, Abcam) and 2 µg/ml anti-HDAC1 (Abcam) antibodies in blocking solution overnight. Visualization was performed using Alexa 680 (Invitrogen) and IRDye800 (Biomol) conjugated secondary antibodies and the Odyssey Infrared Imager (LI-COR).

### ChIP Assay

A549 or NCI-H727 cells (1×10^7^) were seeded into two 75-cm^2^ tissue culture flasks the day before inhibitor treatment. ChIP was performed as described by Bullwinkel et al. [44] 36 h after treatment with 1.5 µM MS-275 (Enzo Life Sciences) or 0.2% DMSO and 24 h after treatment with 400 nM trichostatin A or 0.01% ethanol. Following this protocol, DNA fragments of an average length of approximately 800 bp are produced [44]. The anti-acetyl-Histone H3 (Millipore) and anti-trimethyl-H3K4 (clone MC315, Millipore) antibodies were used for overnight incubation of the precleared samples. Analysis of the isolated DNA was performed by quantitative RT-PCR as described above. The following primers were used: DEFB1 promoter: GAAGAGGGTGAAGTTTGAG, AGGCAGTTCACACTGGA; DEFB1 I: GCGGCAGCCAGATGGAGACAAT, CCCTGGTGTCATTTGCCCTG; DEFB1 II: CCTGCTCAGAGCTTCCCTGT, ACACTGGAGTCCCTCCTTCT; DEFB1 III: CCACTCTGGGTGTCTCATGC, TCACGGTGGTCCAATGAGAA; DEFB1 IV: GACATAGCCTCAGGAAATCC, ATAAAAGCAAGCTGTGCCTT; LINE: GCAGGCCTGGTGGTGAC, CAGAATTTCATATCCAGCCA. Non-specific DNA binding was excluded by subtracting the measured values of a control reaction where no antibody was used.

### Statistical Analysis

Results are expressed as mean ± SD or mean ± SEM as indicated. Analysis of variance of three or more matched groups was performed with the use of the Two-way ANOVA test followed by Bonferroni post-tests. Statistics were performed with GraphPad Prism 5.03 software (GraphPad, San Diego, CA, USA) with differences p<0.05 considered significant. **: p<0.01, ***: p<0.001.
